# Identification of Biotransformation Products of T-2 Toxin in HepG2 Cells Using LC-Q-TOF MS

**DOI:** 10.3390/foods13101501

**Published:** 2024-05-13

**Authors:** Mercedes Taroncher, Veronica Zingales, Yelko Rodríguez-Carrasco, María José Ruiz

**Affiliations:** 1 Laboratory of Food Chemistry and Toxicology, Faculty of Pharmacy and Food Science, University of Valencia, Av. Vicent Andrés Estellés s/n, 46100 Burjassot, Spain; mercedes.taroncher@uv.es (M.T.); veronica.zingales@uv.es (V.Z.); m.jose.ruiz@uv.es (M.J.R.); 2 Research Group Alternative Methods for Determining Toxic Effects and Risk Assessment of Contaminants and Mixtures (RiskTox; GIUV2021-513), University of Valencia, 46100 València, Spain

**Keywords:** T-2, biotransformation, HepG2, in vitro, LC-Q-TOF MS

## Abstract

The T-2 toxin (T-2) is a type A trichothecene found in cereals. The formation of metabolites is a frequent cause of mycotoxin-induced toxicity. In this work, the conversion of T-2 during biotransformation reactions in HepG2 cells was evaluated. For this, HepG2 cells were exposed to 30 (IC_50_/2) and 60 (IC_50_) nM of T-2 for 0, 1, 2, 3, 6, 8 and 24 h, and the concentrations of T-2 and its metabolites HT-2, T2-triol, T2-tetraol and neosolaniol were determined in both the cell fraction and culture medium through liquid chromatography coupled to high-resolution mass spectrometry–time of flight (LC-Q-TOF MS). Results showed a fast metabolization of T-2 (>90%) during the first 2 h, with HT-2 as its main (>95%) biotransformation product. The cell fraction showed higher levels (*p* < 0.05) of HT-2 (39.9 ± 2.1 nM) compared to the culture medium (12.53 ± 2.4 nM). This trend was also observed for the identified metabolites. T2-triol reached its maximum concentration (1.7 ± 0.4 nM) at 2 h, and at later times a time-dependent increase in the T2-tetraol and neosolaniol concentrations was observed. The identification of T-2 metabolites shows the need to continue combined toxicity studies of mycotoxins for a correct risk characterization of these natural contaminants.

## 1. Introduction

Field crops are naturally contaminated by several species of *Fusarium* fungi, which produce trichothecene mycotoxins. These mycotoxins have been frequently evaluated, and the European Food Safety Authority (EFSA) considers that due to toxicity following dietary exposure, there is a potential risk for acute and chronic adverse effects in humans and animals [1]. Therefore, there is a need to identify and quantify their levels in cereals to monitor and verify that the established maximum levels are not exceeded. The T-2 toxin (T-2) is one of the most common mycotoxins of the trichothecenes found mainly in oat, wheat and wheat byproducts [2]. The exposure of T-2 in the population was calculated considering the concentration reported in wheat and wheat-based products and the average consumption of cereals. The maximum level of T-2 found in breakfast cereals for adults and infants was 4.47 μg/kg and 0.11 μg/kg, respectively [1]. Moreover, the wheat and wheat-based products consumption according to data reported by the Food Agriculture Organization was 0.27 kg/day [3]. According to the above, adults are exposed to 2.59 nM T-2, while infants are exposed to 0.064 nM T-2.

As the most toxic trichothecene, T-2 is regarded as the main cause of alimentary toxic aleukia disease (ATA), a gastrointestinal disorder that affected World War II soldiers and people around the world after ingesting moldy food [4]. ATA is characterized by vomiting, diarrhea, gastroenteritis, leukopenia (aleukia), hemorrhages, skin inflammation and, in severe cases, death due to asphyxia [5]. ATA affected some people, especially those between the ages of 10 and 40, with a mortality rate of 60% in the former USSR from 1932 to 1947, mainly due to the large amount of T-2 ingested through grain. However, since then, more analysis on the levels of this mycotoxin in cereals has been carried out and, the maximum levels allowable for the intake of grain without food risk have been determined. Since then, no further cases of ATA have been reported [6,7].

Rapidly after ingestion, T-2 is mainly metabolized into the HT-2 toxin (HT-2) in the liver through a deacetylation reaction at the C-4 position by intestinal microflora, and, in lower concentrations, various phase I and phase II metabolites [8]. It has been previously reported that the main metabolic pathways of T-2 in animals and humans are hydrolysis, hydroxylation, de-epoxidation and glucuronidation [9,10,11]. Furthermore, animal species and gender may affect the metabolic pathways of T-2 [12]. Some of the main T-2 modified forms are HT-2, neosolaniol (Neo), T2-triol, T2-tetraol, 3′-OH-HT-2, 3′-OH-T-2, deepoxy-3′hydroxy-HT-2, deepoxy-3′hydroxy-T-2 triol, deepoxy-15-acetyl-T-2 tetraol and deepoxy T-2 tetraol [13,14]. The main metabolic pathway of T-2 in humans, mice and rats is hydrolysis, and HT-2 is the main metabolite [11]. The toxicity of the metabolites of T-2 is lower than that of T-2 [8,15]. However, the toxicity of HT-2 is very similar to T-2, and their effects cannot be differentiated. The same modified forms were obtained after the metabolism of T-2 and HT-2 [14].

The biotransformation of T-2 results in increased solubility and facilitates excretion of the modified forms, thus reducing the mycotoxin-induced toxicity [12]. However, it should be taken into account that phase II metabolites of T-2 could be hydrolyzed to their parent compounds after intake [16]. Currently, the natural contamination and toxicity of mycotoxin metabolites found in cereals constitute a global health concern. Therefore, it is of great interest to evaluate the biotransformation kinetics and identify the main products of T-2 to make a more accurate evaluation of the real toxicity of the mycotoxin upon ingestion. Hepatic cell lines such as human hepatocarcinoma (HepG2) cells are very appropriate for this evaluation, since they are sensitive to T-2-induced transformation into its modified forms. Moreover, HepG2 cells are widely accepted for the assessment of xenobiotic-induced toxicity and metabolism. It has been reported that HepG2 cells contain phase I and phase II enzymes such as CYP1A, CYP2C9, CYP2E1, CYP2D6 and glutathione-S-transferases [17,18]. Also, Ye and coauthors reported the presence of CYP1A1 in HepG2 cells [19], although the most abundant CYP isoenzyme in HepG2 cells is CYP3A4 [20]. Indeed, Tatay et al. reported that HepG2 cells were able to biotransform some mycotoxins, such as zearalenone (ZEA), into its metabolites [21]. And the metabolic profiling of up to 20 mycotoxins was evaluated in HepG2 cells by Gerdermann et al. [22].

The application of a liquid chromatography coupled to high-resolution mass spectrometry time-of-flight (LC-Q-TOF MS) system allows the identification and quantification of the metabolites in a very accurate manner. This structural elucidation of T-2 and its metabolites HT-2, Neo, T2-triol and T2-tetraol was possible through this combined method. To clarify the metabolism of T-2, in the present study we used HepG2 cells to evaluate its biotransformation products in a cycle of 24 h by identifying and quantifying the T-2 and its main metabolites in both the cell fraction and culture medium. The mycotoxins were selectively detected by LC-Q-TOF MS.

## 2. Materials and Methods

### 2.1. Reagents

The reagent chemicals and cell culture compounds used were as follows: Dulbecco’s Modified Eagle’s Medium (DMEM), trypsin/EDTA solutions, Phosphate Buffer Saline (PBS), penicillin, streptomycin and Newborn Calf Serum (NBCS) were purchased from Sigma-Aldrich (St Louis, MO, USA). Methanol (MeOH) was acquired from Merck Life Science S.L. (Madrid, Spain). Deionized water (resistivity < 18 MΩ cm) was obtained using a Milli-Q water purification system (Millipore, Bedford, MA, USA).

Standards of T-2 (MW:466.52 g/mol), HT-2 (MW:424.48 g/mol) and Neo (MW:382.40 g/mol) were purchased from Sigma-Aldrich, and T2-triol (MW:382.45 g/mol) and T2-tetraol (MW:298.33 g/mol) were acquired from Cayman Chemical Company (St. Louis, MO, USA). Stock solutions of the mycotoxins were prepared in MeOH at appropriate working concentrations and maintained in the dark at −20 °C.

Furthermore, a working standard solution at 10 µM T-2 was prepared to spike the culture medium (3 mL) and reached final mycotoxin concentrations of 30 nM and 60 nM to incubate the cells. All standard solutions were kept in darkness and stored at −20 °C.

### 2.2. Cell Culture and Treatment

HepG2 cells (ATCC: HB-8065) were seeded in DMEM medium supplemented with 100 U/mL penicillin, 10% NBCS and 100 mg/mL streptomycin. A pH of 7.4, 5% CO_2_ at 37 °C and 95% air atmosphere at constant humidity were the incubation conditions. Cells were passaged twice a week with a small number of subpassages (<20 subcultures) to maintain genetic homogeneity. The final mycotoxin concentrations assayed were achieved by adding T-2 to the culture medium with a final MeOH concentration ≤ 1% (*v/v*). Every experiment had appropriate controls with the same volume of solvent.

The T-2 concentrations selected in this study were IC_50_ (60 nM) and IC_50_/2 (30 nM), obtained in previous assays carried out after 24 h of T-2 exposure in HepG2 cells by methyl-thiazol-tetrazolium salt (MTT) [23].

### 2.3. Experimental Design for T-2 Biotransformation Study

HepG2 cells were cultured in 6-well tissue culture plates with 47.2 × 10^4^ cells/well. After 80% of confluence of cells, the medium was replaced with fresh medium containing T-2 at 30 and 60 nM. Cells were exposed to the mycotoxin for 0, 1, 2, 3, 6, 8 and 24 h. Experiments were performed in triplicate at each exposure time. Neither the medium nor the mycotoxin was replaced during the exposure period. After each exposure time, the medium was collected in 15 mL Falcon tubes, 500 μL of trypsin was added to each well and the plates were incubated for 1 min. Then, trypsin-containing cells were transferred to a 5 mL Eppendorf Safe-Lock Microcentrifuge Tube.

### 2.4. Sample Preparation

The extraction of T-2 and its biotransformation products from the medium was carried out following the protocol described by Taroncher et al., 2020 [23]. In summary, a 0.5 mL aliquot of medium was transferred to a 5 mL Eppendorf Safe-Lock Microcentrifuge Tube. Next, 3 mL of ethyl acetate was added, and the mixture was vortexed for 2 min and then centrifuged at 5600× *g* at 4 °C for 5 min (Centrifuge 5810R, Eppendorf, Hamburg, Germany). The collected supernatant was dried under an N_2_ stream at 45 °C with a TurboVap-LV (Zymark, Allschwil, Switzerland). Subsequently, the residue was resuspended in a mixture of 140 µL of MeOH/water (70:30, *v/v*) and vortexed 1 min again before being filtered through a 0.22 µm filter and placed in a vial for analysis.

On the other hand, extraction of the intracellular accumulation of T-2 and/or its biotransformation products was carried out as follows: the medium in the well was removed and 0.5 mL of trypsin was added to the cells. After that, trypsin-containing cells were transferred to a 5 mL Eppendorf Safe-Lock Microcentrifuge Tube. Next, 3 mL of ethyl acetate was added, and the mixture was homogenized with an Ultra-Turrax Ika T18 Basic (Staufen, Germany) for 2 min and subsequently centrifuged at 5600× *g* at 4 °C for 5 min (Centrifuge 5810R, Eppendorf, Hamburg, Germany). The collected supernatant was dried under a nitrogen stream with a TurboVap-LV (Zymark, Allschwil, Switzerland) at 45 °C, resuspended in 140 µL of MeOH/water mixture (70:30, *v/v*) and vortexed for 1 min. Finally, after extracting, it was filtered and placed in a vial for subsequent analysis.

In parallel, T-2 and its metabolites T2-triol, T2-tetraol, HT-2 and Neo were incubated in growth media for 24 h at the same concentrations (30 and 60 nM) and conditions (≤1% MeOH in the medium). The extraction of the analytes was carried out as previously described. These samples were considered as the control and used for recovery studies.

### 2.5. Determination of T-2 and Its Biotransformation Products by LC-Q-TOF MS

The analytical determination was carried out with an LC-Q-TOF MS instrument equipped with an LC Agilent 1200-LC system (Agilent Technologies, Palo Alto, CA, USA) containing a vacuum degasser, an autosampler, a binary pump and a thermostatically controlled column chamber. The chromatographic column comprised a Gemini NX-C18 column (150 × 2 mm, i.d. 3 μm, Phenomenex, Torrance, CA, USA) and a guard column C18 (4 × 2 mm, i.d. 3 μM) kept at 30 °C. The mobile phases were 0.1% formic acid aqueous solution and 5 mM ammonium formate (A) and MeOH with 0.1% of formic acid and 5 mM ammonium formate (B) at a flow rate of 0.200 mL/min. The gradient elution program was as follows: 0–0.5 min, 20% B; 0.5–1 min, 20%~40% B; 1–6 min, 40%~100% B; 6–8 min, 100%~20% B; 8–14 min, 20% B. The injection volume was set to 20 μL.

Mass spectrometry was performed on a 6540 Agilent Ultra-High-Definition Accurate-Mass Quadrupole Time-of-Flight (Q-TOF) MS. The electrospray interface (DUAL AJS ESI) source was operated in positive (ESI^+^) mode. The following comprised the MS parameters set: sheath gas temperature 350 °C at a flow rate of 8 L/min, nebulizer pressure 45 psi, capillary voltage 3.5 kV, drying gas 10 L/min, skimmer voltage 65 V, gas temperature 300 °C, octupole RF peak 750 V and fragmentor voltage 130 V. The scanning range was *m*/*z* 50~1000. The acquisition rate was 3 scans/s for the following collision energies: 10, 20 and 40 eV. Internal mass correction was enabled throughout two reference masses: *m*/*z* 121.050873 and 922.009798. Instrument control and data acquisition were performed using Agilent MassHunter Workstation software B.08.00. Potential analytes were identified using the MassHunter METLIN Metabolite PCD (Personal Compound Database) and PCDL (Personal Compound Database and Library) from Agilent Technologies.

The analyte identification in the target analysis was performed by comparing the retention times of peaks in positive samples with those provided by analytical standards. A strict mass error of 5 ppm was applied to each sample’s matching theoretical mass. Identification points were achieved for suitable confirmatory methods as indicated in the Commission Decision 2002/657/EC. In addition, for a retrospective analysis, characteristics were first extracted from total ion chromatograms using the batch recursive feature extraction algorithm for small molecules using Agilent MassHunter Profinder (Agilent Technologies). The settings for this algorithm were as follows: retention time filter, 1–14 min; ion intensity filter, 600 counts; retention time tolerance, ±0.3 min; mass tolerance, ±5 ppm; Q-score > 80. Following peak deconvolution and chromatographic peak alignment, the MassHunter Mass Profiler software B.08.00 and the Agilent Mycotoxins and Related Metabolites PCDL were used to identify the extracted features. The following criteria were used to identify the features: mass tolerance ±5 ppm; positive ions included H^+^, NH_4_^+^; mass score, 100; isotope abundance score, 60; isotope spacing score, 50.

### 2.6. Method Validation

For the quantitative analysis of T-2 and its biotransformation products, the method was fully validated according to Commission Decision 2002/657/EC concerning the performance of analytical methods. Selectivity was evaluated by using blank culture medium samples. Responses attributable to interfering components should not be more than 20% of the analyte’s response at the limit of quantification (LOQ). The LOQ was defined as the lowest concentration within the linear range at which the instrument was capable of quantifying the analyte with a mass error below 5 ppm. The carryover effect was evaluated by injecting blank samples after the highest calibration points of the calibration curves. The calibration curves were constructed using a linear regression model in a linear range from LOQ to 120 ng/mL, and at least six calibration standard levels should meet the acceptance criteria. The concentration levels of the calibration curves were analyzed to evaluate the accuracy and precision in five replicates within three validation batches. The signal suppression–enhancement (SSE) was evaluated by comparing signal intensities from blank samples spiked at 30 nM after extraction with those obtained from injection of neat standard solutions at the same concentration dissolved in the injection solvent. An SSE value of 100% indicates that no matrix effect was present. SSE values below 100% indicate signal suppression, and values above 100% indicate signal enhancement. The accuracy and precision were assessed in triplicate (repeatability, RSD_r_) on three different days (reproducibility, RSD_R_). The recovery was determined by comparing the analyte responses in blank and spiked samples at 30 and 60 nM of each mycotoxin. The precision was assessed by calculating the relative standard deviation (RSD).

Data from mass spectrometry and chromatography were used to confirm the presence of analytes in the samples. Therefore, mycotoxin identification was carried out at each of the following identification points: the exact mass set to five decimal places and the retention time from the extracted ion chromatogram of the peak in samples compared with those of standard solutions at a tolerance of ±2.5% were used to confirm the peaks for the mycotoxins included in this study. The mass accuracy (∆) for a measured ion was calculated according to the following formula and reported as part per million (ppm):

(1) $$\text{∆}\left(\mathrm{ppm}\right)=1\times 10^{6}\frac{\left(m/z_{\mathrm{measured}}-m/z_{\mathrm{theoretical}}\right)}{m/z_{\mathrm{theoretical}}}$$

The effectiveness of the validated method was demonstrated by including a blank sample, a reagent blank, a replicate sample and the lowest calibration point at the beginning and at the end of each group of samples for quality assurance/quality control (QA/QC) analysis. Samples were analyzed in duplicate, and quantifiable values of mycotoxins should be identified in both replicates for confirmatory purposes.

### 2.7. Statistical Analysis

The statistical software Statgraphics version 16.01.03 (IBM Corp., Armonk, NY, USA) was used to analyze the data statistically. Three independent experiments’ mean ± standard error of the mean (SEM) were used to express the data. The Student *t*-test for paired samples was used to statistically analyze the data. The one-way analysis of variance (ANOVA) followed by a Tukey HDS post hoc test for multiple comparisons were used to analyze differences between groups. Statistical significance was considered as *p* ≤ 0.05.

## 3. Results and Discussion

### 3.1. Optimization of Q-TOF HRMS Parameters

By injecting the analytical standards of T-2, HT-2, T2-triol, T2-tetraol and Neo at a concentration of 1 µg/mL, the compound-dependent MS parameters were optimized. The instrument operated in both ionization modes in order to determine the ionization mode that generated better ionization of the analytes under study. To evaluate the accuracy of the mass measurements, they were also compared to the theoretical masses. The studied mycotoxins formed stable ammonium and protonated adducts, and therefore, they were monitored to evaluate the fragments that generated greater intensity for subsequent analysis. Table 1 shows the analytical parameters of the studied compounds, including elemental composition, retention time, adduct ion, measured mass, mass accuracy and fragmentation pattern. Analytes were eluted from 8.84 to 11.62 min. As expected, T-2 metabolites were eluted first, meaning they showed a more polar character, and they are considered as detoxification products of a parent compound, in agreement with previous studies [10]. When compared to the theoretical masses, the chosen ions showed excellent accuracy, with mass errors within a strict range (<2 ppm). For T-2, HT-2 and Neo, the characteristic ammonium adducts [M + NH_4_]^+^ showed the highest intensity, whereas the protonated ions [M + H]^+^ for T2-triol and T2-tetraol were chosen based on the displayed intensities.

### 3.2. Method Performance

Table 2 summarizes the results of the method validation. The signal response of the investigated MS, in an interval of six points between the LOQ and 120 ng/mL, was linear for all analytes, obtaining a correlation coefficient greater than 0.990 for the evaluated mycotoxins. Regarding the matrix effect, all analytes showed an absolute value of matrix effect greater than 90%. These results indicated that the culture medium barely influenced the response of the analytes, and these could be quantified through a calibration curve built in solvent. The accuracy results showed that the extraction of the analytes was optimal at the two concentration levels tested, obtaining a recovery range from 80.4 to 84.1% at 30 nM and from 87.3 to 94.6% at 60 nM. Likewise, the method was repeatable and reproducible, showing RSD_r_ and RSD_R_ values lower than 10% and 12%, respectively, in all cases. The LOQs obtained showed good sensitivity, being able to quantify concentrations of the assayed mycotoxins between 0.02 and 0.2 ng/mL.

### 3.3. Identification of T-2 and Its Metabolites

To study the biotransformation of T-2 by HepG2 cells, the cells were exposed to 30 and 60 nM of T-2 for a time of 0, 1, 2, 3, 6, 8 and 24 h. Each assay was carried out in triplicate, and after each exposure time, the culture medium was collected. Similarly, the cells contained in the wells were redissolved in 0.5 mL of trypsin, and these cell fractions were taken to evaluate the intracellular accumulation of T-2 and/or its biotransformation products. The extraction and determination of T-2 and its metabolites in both the culture medium and the cells were carried out as previously mentioned.

The results revealed a rapid and complete T-2 biotransformation based on the mass shift of metabolites obtained from MS full scan and MS/MS. Metabolites are typically slightly different from those of the parent compound due to metabolic transformation pathways known as oxidation by enzymes in microsomal cytochromes P450, reduction by epoxide hydrolases and hydrolysis by nonspecific esterases and amidases. Hydroxylation has been well characterized and is catalyzed by CYP450 enzymes [24].

**Figure 1:**
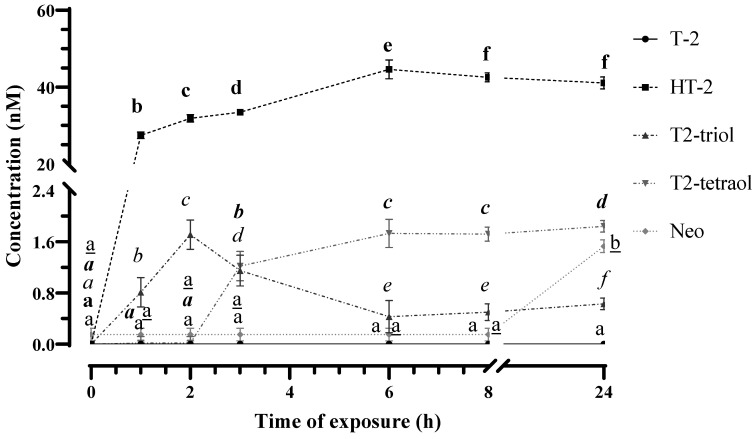
Concentrations of T-2, HT-2, T2-triol, T2-tetraol and Neo identified and quantified by LC-Q-TOF MS in the cell fraction of HepG2 cells after 0, 1, 2, 3, 6, 8 and 24 h of 60 nM of T-2 exposure. Values are expressed as mean ± SEM (n = 3). Values with different superscript letters for each metabolite are significantly different (*p* ≤ 0.05). The black superscript letter indicates the statistic of T-2. The bold superscript letter indicates the statistic of HT-2. The italic superscript letter indicates the statistic of T2-triol. The bold italic superscript letter indicates the statistic of T2-tetraol. The underlined superscript letter indicates the statistic of Neo.

**Figure 2:**
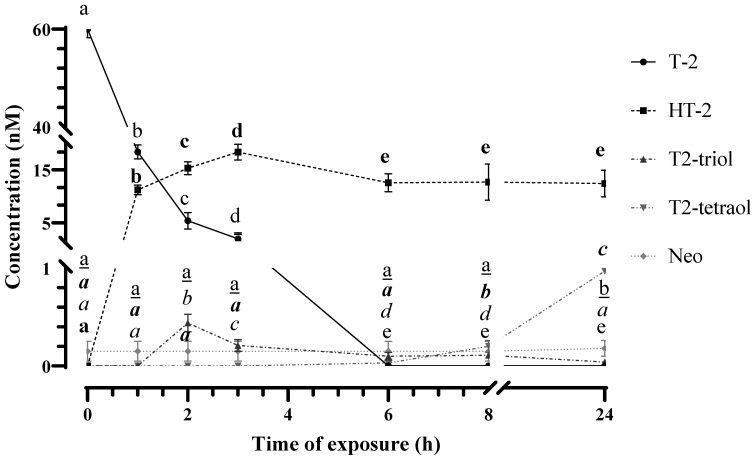
Concentrations of T-2, HT-2, T2-triol, T2-tetraol and Neo identified and quantified by LC-Q-TOF MS in the culture medium of HepG2 cells after 0, 1, 2, 3, 6, 8 and 24 h of 60 nM of T-2 exposure. Values are expressed as mean ± SEM (n = 3). Values with different superscript letters for each metabolite are significantly different (*p* ≤ 0.05). The black superscript letter indicates the statistic of T-2. The bold superscript letter indicates the statistic of HT-2. The italic superscript letter indicates the statistic of T2-triol. The bold italic superscript letter indicates the statistic of T2-tetraol. The underlined superscript letter indicates the statistic of Neo.

Despite these modifications, during collision-induced dissociation, they still retain a common skeleton, resulting in similar fragmentation patterns. Consequently, the chemical structures of T-2 metabolites were evaluated by analyzing the mass change and fragmentation pattern obtained from the full MS scan and MS/MS analysis. Figure 1 and Figure 2 and Tables S1 and S2 show the evolution of T-2 levels (60 nM) and T-2′s biotransformation products in both the HepG2 cellular fraction and the cultured medium, respectively, collected at different assayed times.

**Figure 3:**
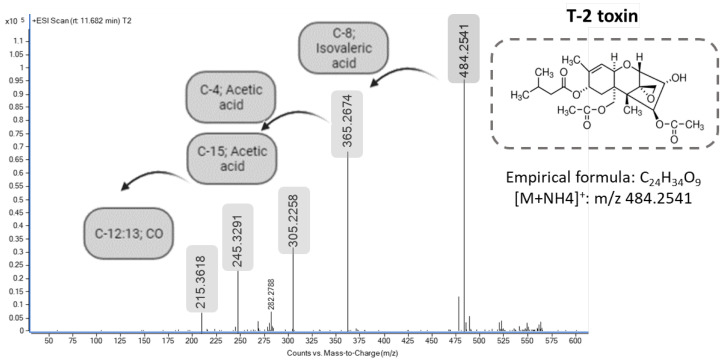
Mass spectra of T-2 standard. Precursor ion at *m*/*z* 484.2541 ([H + NH_4_]^+^) and major product ions at *m*/*z* 365.2674 and *m*/*z* 205.2258.

At time 0 h, after comparing the signal obtained from the MS full scan and MS/MS analysis with the mycotoxin standards, it was found that the observed peak had the same retention time (Rt = 11.62 min), accurate MS value (ammonium adduct [M + NH_4_]^+^ at *m*/*z* 484.2539, −0.81 ppm) and MS/MS fragmentation pattern as the T-2 standard. In fact, the MS/MS spectra of the observed peak generated product ions at *m*/*z* 305 due to the elimination of the acetic acid side chain (42 Da) in position C-4 and at *m*/*z* 365 because of the breakage of the isovaleric acid side chain at the C-8 position and a neutral loss of NH_3_·H_2_O (119 Da) (Figure 3). Therefore, T-2 was the only mycotoxin identified at 0 h in the culture medium and quantified at 58.7 ± 1.7 nM, and none of its metabolites were detected in the medium or inside the cell, probably due to the short exposure time that was not sufficient for its metabolization.

**Figure 4:**
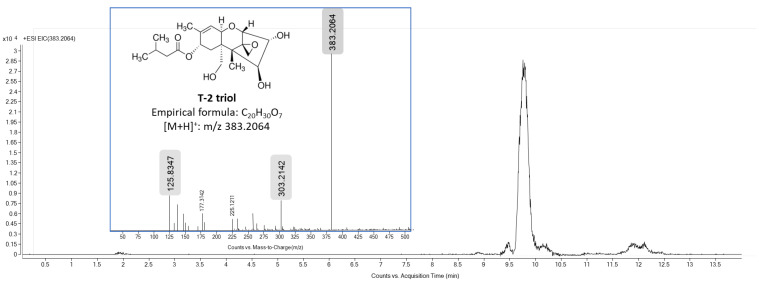
Total ion chromatogram (*m*/*z* 383.2064 [M + H]^+^) and mass spectra of T2-triol standard. The major product ion peak was obtained at *m*/*z* 303.2142.

Nonetheless, after 1 h of exposure, the T-2 concentration found in the culture medium was significantly lower (18.3 ± 1.3 nM) compared to the initial time, as T-2 was not detected in the cell fraction. In addition, at this exposure time, another signal was detected at 10.87 min (ammonium adduct [M + NH_4_]^+^ at *m*/*z* 442.2435, −1.26 ppm) both in the culture medium and in the cell fraction. The MS/MS spectra of this signal generated fragment ions at *m*/*z* 323 by the cleavage of the isovaleric acid side chain at the C-8 position and the C-3 or -4 position (H_2_O) and at *m*/*z* 263, which is formed from *m*/*z* 323 by the loss of the acetyl group and H_2_O (60 Da) at the C-15 position. Hence, this mycotoxin was identified as HT-2, as the abovementioned data were similar when compared with the HT-2 analytical standard. HT-2 was quantified at 11.2 ± 2.1 nM and 27.4 ± 3.5 nM in the culture medium and cell fraction, respectively, after 1 h of exposure. In addition, a weak signal was also detected at 9.75 min, which is its accurate MS value (protonated adduct [M + H]^+^ at *m*/*z* 383.2064, −1.02 ppm), and ab MS/MS fragmentation pattern similar to that of T2-triol’s analytical standard (Figure 4). Hence, T2-triol (also known as deacetyl HT-2 toxin) was only detected at 0.82 ± 0.2 nM in the cell fraction after 1 h of T-2 exposure.

**Figure 5:**
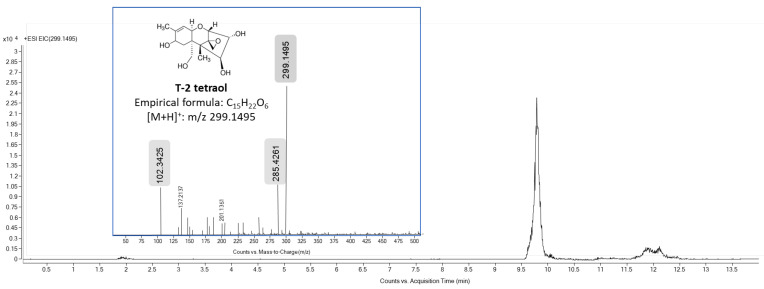
Total ion chromatogram (*m*/*z* 299.1495 [M + H]^+^) and mass spectra of T2-tretraol standard. The major product ion peak was obtained at *m*/*z* 285.4261.

Following 2 and 3 h of T-2 exposure to HepG2 cells, a significant decrease in the mycotoxin concentration in the culture medium was again observed (5.5 ± 0.9 nM after 2 h and 1.9 ± 0.3 nM after 3 h of exposure). This reduction evidenced the biotransformation of T-2 into its metabolites HT-2 and T2-triol. In detail, an increase (*p* < 0.05) in the concentration of HT-2 was observed in both the cell fraction (32.5 ± 2.8 nM at 2 h and 36.0 ± 3.1 nM at 3 h) and in the culture medium (15.6 ± 2.0 nM at 2 h and 18.2 ± 3.4 nM at 3 h). About T2-triol, a significant reduction in the concentration of this metabolite was observed in both the cell fraction and the culture medium between these investigated times (1.7 ± 0.4 nM versus 1.1 ± 0.3 nM in cell fraction and 0.5 ± 0.1 nM vs. 0.2 ± 0.03 nM in culture medium at 2 and 3 h, respectively). It is noteworthy that after 3 h of exposure, a slight signal was additionally detected at 9.91 min in samples from the cell fraction, which is its accurate MS value (protonated adduct [M + H]^+^ at *m*/*z* 299.1495, −0.54 ppm), and the MS/MS fragmentation pattern coincident with the values obtained from the T2-tetraol analytical standard (Figure 5). Thus, the reduction in T2-triol levels could be due to its biotransformation to T2-tetraol since this can be formed after the dealkylation of T2-triol or deacetylation of neosolaniol-derived metabolites, as previously reported by Yang et al. [13].

After 6 h of exposure, T-2 was no longer detected in the culture medium. As far as its main metabolite is concerned, a reduction (*p* < 0.05) in HT-2 concentration was quantified, with no significant changes observed in its levels between 6 h and 24 h (12.53 ± 2.4 nM) of exposure. In parallel, a significant increase in HT-2 levels was observed in the cell fraction at 6 h (44.4 ± 3.9 nM) and subsequently a reduction, with no differences (*p* < 0.05) between 8 h and 24 h (39.9 ± 2.1 nM). Concerning T2-triol, the levels found in the culture medium were significantly reduced throughout the exposure time and were not detected after 24 h. Similarly, in the cell fraction, a reduction (*p* < 0.05) in levels of this metabolite was observed between 3 h (1.1 ± 0.6 nM) and 6 h (0.4 ± 0.07 nM) of exposure, and subsequently the levels fluctuated up to a concentration of 0.64 ± 0.12 nM at 24 h of exposure. These fluctuations could be justified considering the results observed for the toxin T2-tetraol, since a significant increase in its concentration was observed as the exposure time increased from 3 h (1.20 ± 0.3 nM) to 24 h in the cell fraction (1.8 ± 0.7 nM). Regarding the culture medium, T2-tetraol was detected for the first time at 6 h of exposure (0.03 ± 0.01 nM), and its concentration increased significantly until 24 h of exposure (1.05 ± 0.03 nM).

**Figure 6:**
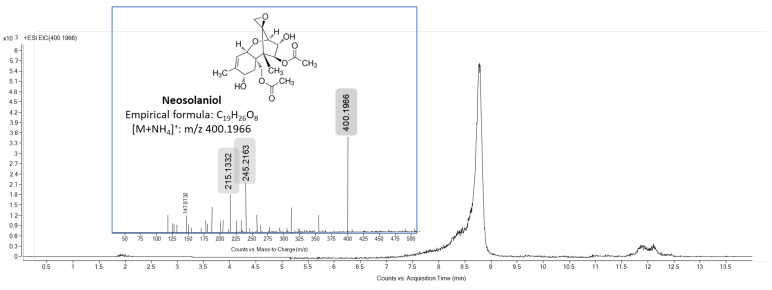
Total ion chromatogram (*m*/*z* 400.1966 [M + NH_4_]^+^) and mass spectra of Neo standard. The major product ion peak was obtained at *m*/*z* 245.2163.

Finally, after 24 h of exposure, a new but weak signal was detected in both the culture medium and the cell fraction. This metabolite was eluted at 8.84 min and showed a [M + NH_4_]^+^ ion at *m*/*z* 400.1966 (−1.21 ppm) with the elemental composition of C_19_H_30_NO_8_, suggesting that it was a metabolite of T-2 from the cleavage of an isovaleryl at the C-8 position. The MS/MS spectra of this signal generated fragment ions at *m*/*z* 245 and 215, which correspond to [T2 − isoval acid − 2 acetic acid + H]^+^ (sum formula neutral compound C_15_H_16_O_3_) and [T2 − isoval acid − 2 acetic acid − CH_2_O + H]^+^ (sum formula neutral compound C_14_H_14_O_2_), respectively (Figure 6). These MS and MS/MS values were coincident with the values of the Neo analytical standard. Hence, Neo was quantified at 0.2 ± 0.02 nM and at 1.54 ± 0.61 nM in culture medium and cell fraction, respectively, after 24 h of T-2 exposure in HepG2 cells. This metabolite could be further metabolized to 4-deacetyl-neosolaniol and 15-deacetyl-neosolaniol and then to T2-tetraol after deacetylation, as also evidenced Yang et al. [25].

In addition, after total ion chromatograms were properly processed to eliminate unnecessary information and produce accurate findings, a retrospective analysis of samples based on the Related Metabolites PCDL and the Agilent Mycotoxins spectrum library was carried out. The results showed that additional metabolites were putatively identified in both culture medium and cell fraction samples collected after 6 h of exposure to T-2. In detail, weak signals produced peaks at 9.45 min (*m*/*z* 500.2487) and at 9.21 min (*m*/*z* 458.2371). These ions were observed to have a mass 16 Da higher than T-2 and HT-2, respectively. This suggests that they were monohydroxylated forms of T-2 (C_24_H_38_NO_10_^+^) and HT-2 (C_22_H_36_NO_9_^+^). Based on previous research on T-2 biotransformation products, these metabolites were identified to be 3′-OH-T-2 and 3′-OH-HT-2, respectively [13,26]. However, these signals were weak in the detected samples and could not be confirmed due to the lack of analytical standards.

T-2 metabolism is very complex because there are many metabolites detected and the CYPs involved depend on the species. According to Lootens et al., during the hydroxylation of the T-2 toxin in the liver, the CYP450s play an important role in the process of T-2 biotransformation, and the CYPs involved differ among different species of animals. The main CYP enzymes involved in the metabolism of T-2 are CYP3A22, CYP3A29 and CYP3A46 in pigs [27]; CYP1A4, CYP1A5, CYP2C18 and CYP3A37 in chickens [28]; and CYP1A1, CYP1A2, CYP2A1 and CYP2B4 in rabbits [29]. On the other hand, Lin et al. reported that the contributions of the CYP450 enzyme family to T-2 metabolism in vitro followed the descending order of CYP3A4, CYP2E1, CYP1A2, CYP2B6 or CYP2D6 or CYP2C19 [30]. However, a variety of results have also been reported depending on the cell species. For example, the enzyme that plays the main role in the metabolism of T-2 is CYP1A1 in Caco-2 cells [31], CYP3A4 and CYP1A1 in HepG2 cells [15,24,32,33] and CYP3A46 in porcine primary hepatocytes [34].

**Figure 7:**
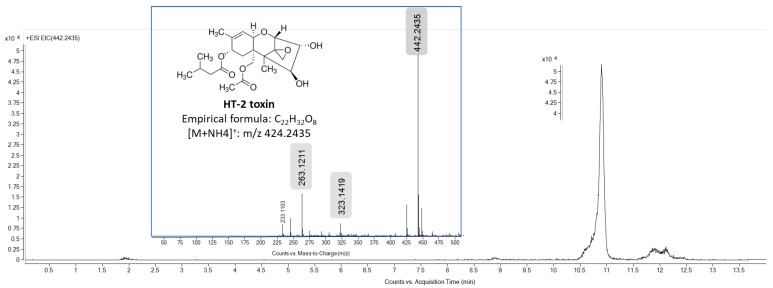
Total ion chromatogram (*m*/*z* 442.2435 [M + NH_4_]^+^) and mass spectra of HT-2 standard. The major product ion peak was obtained at *m*/*z* 263.1211.

Regarding the results obtained after exposing HepG2 cells to 30 nM of T-2 and studying their biotransformation from 0 to 24 h, it should be noted that only HT-2 was detected in quantifiable amounts (from LOQ to 17.9 ± 2.3 nM) (Figure 7). The evolution of HT-2 levels reproduces the trend observed in this study carried out with 60 nM of T-2. The rest of the metabolites identified in this targeted and retrospective study at 60 nM of T-2 were not detected at 30 nM of T-2. This is probably due to low levels that would be below the LOQ of the method, despite showing good sensitivity. This T-2 concentration corresponds to the IC_50_/2 value according to other studies [15]. Conducting studies at low doses of T-2 is appropriate given the levels of mycotoxin contamination that typically appear in foods. Even at these doses, the toxic effects are notable, which could involve activation of oxidative stress, mitochondrial damage, abnormal DNA methylation, autophagy and apoptosis, as recently reviewed in the literature [33,35]. This is also highlighted by the results of a study carried out at low doses of T-2 (50% LOAEL); they found that T-2 reduces the synthesis of anti-inflammatory cytokines such as TGF-β and increases the secretion of proinflammatory cytokines, compromising the integrity of the intestinal mucosal barrier [36]. Furthermore, combined toxic effects due to the coexposure of T-2 and its metabolites should also be considered because these combinations exhibit varying degrees of cytotoxicity compared to mycotoxins ingested individually, with stronger cytotoxicity, as recently reviewed by Skrzydlewski and colleagues [37].

The findings on the T-2 biotransformation products over 24 h help to fill the gap in the field of trichothecenes. They contribute to providing further information needed to update the established maximum levels of T-2 exposure based on the expected toxicity of this mycotoxin and its metabolites and the possible effects of synergism or interaction between them. In this way, a better risk assessment is carried out, given that in recent years, the EFSA has considered T-2 toxicity to be ended, but this has been reassessed.

## 4. Conclusions

A sensitive LC-QTOF MS method was applied for the structural elucidation of T-2 biotransformation products formation over 24 h in an in vitro system. The T-2 was rapidly metabolized by HepG2 cells, and its hydroxylation metabolites were quantified, with HT-2, T2-triol, T2-tetraol and Neo as the main metabolites. Through the combination of accurate mass measurements providing qualitative information of TOF and the MassHunter Mass Profiler software, additional metabolites were putatively identified. Additional investigation is required to comprehend the molecular processes behind the cytotoxicity of several mycotoxins in animals and humans. This includes delving into the whole route and alterations of important enzymes in the mycotoxins pathway.

The next step in the research is to study the toxicity produced by the mixture of these mycotoxins. In the future, we intend to make binary and tertiary combinations and to test their possible synergistic, additive or antagonistic effects. Furthermore, with in silico computational methods, we hope to establish which are the target organs of these mycotoxins in order to model the possible toxicity in 3D to carry out assays in hepatic spheroids.

## Figures and Tables

**Table 1:** LC-Q-TOF MS parameters for the target mycotoxins.

Compound	Retention Time (min)	Molecular Formula	Measured Mass (*m*/*z*)	Adduct	Mass Accuarcy (Δppm)	Major Fragments
T-2	11.62	C_24_H_34_O_9_	484.2541	[M + NH_4_]^+^	−0.81	365 ^a^, 305
HT-2	10.87	C_22_H_32_O_8_	442.2435	[M + NH_4_]^+^	−1.26	263 ^a^, 323
T2-triol	9.75	C_20_H_30_O_7_	383.2064	[M + H]^+^	−1.02	303 ^a^, 125
T2-tetraol	9.91	C_15_H_22_O_6_	299.1495	[M + H]^+^	−0.54	285 ^a^, 102
Neo	8.84	C_19_H_26_O_8_	400.1966	[M + NH_4_]^+^	−1.21	215 ^a^, 245

^a^ The base peak in the MS/MS spectra.

**Table 2:** Method performance.

		Recovery (%)		Precision (%) [RSD_r_ ^b^, (RSD_R_ ^c^)]		
Analyte	SSE ^a^ (%)	30 nM	60 nM	30 nM	60 nM	LOQ ^d^ (ng/mL)
T-2	95	84.1	94.6	6.3 (9.2)	5.4 (8.2)	0.05
HT-2	97	82.0	90.8	7.2 (9.5)	6.3 (7.6)	0.10
T2-triol	91	83.1	90.3	6.7 (10.3)	7.2 (9.7)	0.04
T2-tetraol	90	80.9	89.7	6.4 (10.5)	8.6 (10.8)	0.02
Neo	94	80.4	87.3	9.1 (11.7)	5.9 (9.4)	0.20

^a^ SSE: signal suppression–enhancement; ^b^ RSD_r_: repeatability; ^c^ RSD_R_: reproducibility; ^d^ LOQ: limit of quantification.

## Data Availability

The original contributions presented in the study are included in the article, further inquiries can be directed to the corresponding author.

## Supplementary Materials

The following supporting information can be downloaded at: https://www.mdpi.com/article/10.3390/foods13101501/s1, Table S1: Concentration of T-2, HT-2, T2-triol, T2-tetraol and Neo identified and quantified by LC-Q-TOF MS in the cell fraction of HepG2 cells after 0, 1, 2, 3, 6, 8 and 24 h of 60 nM of T-2 exposure to 60 nM T-2; Table S2: Concentration of T-2, HT-2, T2-triol, T2-tetraol and Neo identified and quantified by LC-Q-TOF MS in the culture medium of HepG2 cells after 0, 1, 2, 3, 6, 8 and 24 h of 60 nM of T-2 exposure.
