# Supplementary material for: Identification of Biotransformation Products of T-2 Toxin in HepG2 Cells Using LC-Q-TOF MS
Source: Foods. 2024 May 13;13(10):1501. doi: 10.3390/foods13101501 (PMC11120489; doi:10.3390/foods13101501)
Supplement: Supplementary file 1 [file foods-13-01501-s001.zip › foods-2995238-supplementary.pdf]

SUPLEMENTARY MATERIAL

Identification of Biotransformation Products of T-2 Toxin in HepG2 Cells Using LC-Q-TOF MS

Mercedes Taroncher [1,2], Veronica Zingales [1,2], Yelko Rodríguez-Carrasco [1,2,*] and María José Ruiz [1,2]

[1] Laboratory of Food Chemistry and Toxicology, Faculty of Pharmacy and Food Science, University of Valencia, Av. Vicent Andrés Estellés s/n, 46100 Burjassot, Spain; mercedes.taroncher@uv.es (M.T.); veronica.zingales@uv.es (V.Z.); m.jose.ruiz@uv.es (M.J.R.)

[2] Research Group Alternative Methods for Determining Toxic Effects and Risk Assessment of Contaminants and Mixtures (RiskTox; GIUV2021-513), University of Valencia, 46100 València, Spain

* Correspondence: yelko.rodriguez@uv.es; Tel./Fax: +34-96-3544-228

CELL FRACTION					
**Time of exposure**	**Mycotoxin**				
	**T-2 (nM)**	**HT-2 (nM)**	**T-2 triol (nM)**	**T-2 tetraol (nM)**	**NEO (nM)**
**0h**	<LOQ	<LOQ	<LOQ	<LOQ	<LOQ
	<LOQ	<LOQ	<LOQ	<LOQ	<LOQ
	<LOQ	<LOQ	<LOQ	<LOQ	<LOQ
**1h**	<LOQ	27,43	0,72	<LOQ	<LOQ
	<LOQ	25,6	0,94	<LOQ	<LOQ
	<LOQ	24,8	0,8	<LOQ	<LOQ
**2h**	<LOQ	32	1,65	<LOQ	<LOQ
	<LOQ	31,87	1,64	<LOQ	<LOQ
	<LOQ	33,51	1,7	<LOQ	<LOQ
**3h**	<LOQ	33,56	1,11	1,19	<LOQ
	<LOQ	34,84	1,17	1,24	<LOQ
	<LOQ	39,54	1,16	1,18	<LOQ
**6h**	<LOQ	44,41	0,39	1,74	<LOQ
	<LOQ	43,61	0,34	1,81	<LOQ
	<LOQ	45,17	0,42	1,73	<LOQ
**8h**	<LOQ	40,61	0,51	1,71	<LOQ
	<LOQ	41,53	0,52	1,73	<LOQ
	<LOQ	40,58	0,55	1,74	<LOQ
**24h**	<LOQ	39,47	0,61	1,84	1,46
	<LOQ	38,84	0,63	1,86	1,52
	<LOQ	41,61	0,68	1,85	1,63

Table S1. *Concentration of T-2, HT-2, T2-triol, T2-tetraol and Neo identified and quantified by LC-Q-TOF MS in the cell fraction of HepG2 cells after 0, 1, 2, 3, 6, 8 and 24 h of 60 nM of T-2 exposure to 60 nM T-2.*

CULTURE MEDIUM					
**Time of exposure**	**Mycotoxin**				
	**T-2 (nM)**	**HT-2 (nM)**	**T-2 triol (nM)**	**T-2 tetraol (nM)**	**NEO (nM)**
**0h**	57,8	<LOQ	<LOQ	<LOQ	<LOQ
	59,4	<LOQ	<LOQ	<LOQ	<LOQ
	58,9	<LOQ	<LOQ	<LOQ	<LOQ
**1h**	18,4	11,3	<LOQ	<LOQ	<LOQ
	17,6	11,1	<LOQ	<LOQ	<LOQ
	18,9	11,5	<LOQ	<LOQ	<LOQ
**2h**	5,4	15,4	0,41	<LOQ	<LOQ
	5,3	15,7	0,46	<LOQ	<LOQ
	5,8	15,6	0,51	<LOQ	<LOQ
**3h**	1,7	18,1	0,19	<LOQ	<LOQ
	1,8	17,9	0,24	<LOQ	<LOQ
	2,1	18,5	0,23	<LOQ	<LOQ
**6h**	<LOQ	12,4	0,15	0,02	<LOQ
	<LOQ	12,6	0,12	0,05	<LOQ
	<LOQ	12,7	0,09	0,03	<LOQ
**8h**	<LOQ	12,7	0,11	0,21	<LOQ
	<LOQ	12,6	0,14	0,19	<LOQ
	<LOQ	12,4	0,08	0,23	<LOQ
**24h**	<LOQ	12,6	0,03	0,94	0,21
	<LOQ	12,1	0,02	1,23	0,22
	<LOQ	12,9	0,05	0,98	0,17

Table S2. *Concentration of T-2, HT-2, T2-triol, T2-tetraol and Neo identified and quantified by LC-Q-TOF MS in the culture medium of HepG2 cells after 0, 1, 2, 3, 6, 8 and 24 h of 60 nM of T-2 exposure.*
